# Pesticides Drive Liver Diseases Through Non-Apoptotic Regulated Cell Death Pathways

**DOI:** 10.3390/diseases14030096

**Published:** 2026-03-05

**Authors:** Zamza Khairullina, Saulesh Kurmangaliyeva, Rustam Yussupov, Elmira Kelimberdiyeva, Liliya Tryfonyuk, Nasriddin Shapambayev, Aizat Seidakhmetova, Talgat Medetbekov, Anton Tkachenko

**Affiliations:** 1 Department of Microbiology, Virology and Immunology, West Kazakhstan Marat Ospanov Medical University, 68 Maresyev St., Aktobe 030000, Kazakhstan; 2 Department of Microbiology, Virology and Immunology, Asfendiyarov Kazakh National Medical University, 94 Tole bi St., Almaty 050000, Kazakhstan; 3 Department of General Medical Practice, Astana Medical University, 49/A Beibitshilik St., Astana 010000, Kazakhstan; 4 Institute of Health, National University of Water and Environmental Engineering, 11 Soborna St., 33028 Rivne, Ukraine; 5 Rivne Regional Clinical Hospital, 78g Kyivska St., 33007 Rivne, Ukraine; 6 Department of General Practitioner No. 1, Khoja Akhmet Yasawi International Kazakh-Turkish University, 7/7 Baitursynov St., Shymkent 160012, Kazakhstan; 7 Department of Emergency Medicine and Nursing, South Kazakhstan Medical Academy, 1/1 Al-Farabi Sq., Shymkent 160019, Kazakhstan; 8 Department of Surgical Diseases No. 2, Asfendiyarov Kazakh National Medical University, 94 Tole bi St., Almaty 050000, Kazakhstan; 9 BIOCEV, First Faculty of Medicine, Charles University, Průmyslová 595, 25250 Vestec, Czech Republic

**Keywords:** apoptosis, damage-associated molecular patterns, ferroptosis, necroptosis, pyroptosis, regulated cell death

## Abstract

A compelling body of evidence links pesticide exposure to human diseases. The liver plays a central role in the detoxification of pesticides, suggesting intense pesticide–liver cell interactions. A growing body of studies highlighted in this review supports the contribution of pesticides of various chemical classes to the development of non-alcoholic fatty liver disease (NAFLD), alcohol-associated liver disease (ALD), liver cirrhosis, viral hepatitis, hepatocellular carcinoma, etc., via disrupting lipid and carbohydrate metabolism and redox homeostasis, promoting endoplasmic reticulum stress and mitochondrial dysfunction, as well as stimulating apoptosis, fibrosis, and inflammation. In this review, we systematically illustrated an underappreciated mechanism of pesticide-induced overall and hepatic toxicity, i.e., the ability to induce non-apoptotic regulated cell death (RCD) pathways such as ferroptosis, necroptosis, and pyroptosis. Our analysis indicates that pesticides are implicated in driving liver diseases by inducing ferroptosis, necroptosis, and pyroptosis. Non-apoptotic RCDs mediate pesticide-induced liver steatosis and fibrosis. Furthermore, these cell death modalities fuel inflammation through the promotion of pro-inflammatory cytokine production and the generation of damage-associated molecular patterns. Understanding of deeper mechanisms of pesticide-induced effects on the non-apoptotic cell death machinery and subsequent immunogenic effects in liver pathology might help develop novel preventive strategies to reduce liver damage.

## 1. Introduction

Pesticides are currently widely used in agriculture to combat undesirable pests, e.g., rodents, weeds, insects, fungi, or microorganisms, to increase the yield and quality of crops [1,2]. Based on their targets, pesticides can be subdivided into rodenticides (used against rodents), herbicides (weeds), insecticides (insects), fungicides (fungi), nematicides (nematodes), acaricides (mites and ticks), and bactericides (bacteria) [3,4]. Over recent decades, pesticides have emerged as an important tool to boost agricultural productivity. Therefore, their production and agricultural application have skyrocketed. There is compelling evidence that their effectiveness for increasing crop output has been linked to their excessive use, which contributes to environmental pollution and the associated enhanced exposure of humans and animals [4]. Annual sales of pesticides in the European Union reach approximately 350,000 tonnes [5]. In 2019, the total pesticide market size was estimated to approach $85 billion [6]. The constant growth of this market is emphasized by the fact that its size is expected to increase to $280 billion by 2030 [7]. It is important to note that the social and economic burden of the inappropriate pesticide use in crop production is indeed high. According to recent estimates performed by Rufo et al. in their meta-analysis, the global cost of pesticide use is assessed to be over $50 per individual [8]. Thus, although pesticides enhance agricultural yields, their use is linked to certain disadvantages and limitations.

Due to the abundant environmental accumulation of pesticides worldwide, their safety raises concerns, and the complex investigation of their toxicological effects in humans is prioritized. Converging lines of evidence indicate that exposure to pesticides is linked to a wide range of adverse effects [9,10]. In particular, pesticides have been shown to exert neurotoxic effects, contributing to the emergence of neurodegenerative disorders, e.g., Alzheimer’s disease, Parkinson’s disease, etc. [11,12]. Furthermore, pesticide consumption has been associated with an increased risk of cardiovascular pathology [13,14], for instance, ischemic heart disease [15]. Importantly, the impact of pesticides on cardiovascular health is variable and might be dependent on the chemical group of pesticides [16]. At the same time, variability in effects is also observed within the same group of pesticides. Environmental and occupational exposure to pesticides has been reported to alter pulmonary health, correlating with the development of lung cancer, asthma, or chronic obstructive pulmonary disease [17,18,19]. Likewise, human exposure to pesticides increases the probability of developing celiac disease and irritable bowel syndrome [20]. Negative effects of pesticides on the gut have been attributed to modifications of the gut microbiota [21,22]. Moreover, there is some evidence concerning the links between pesticide-induced changes in the intestinal microbiome and behavior, since behavioral features are regulated by the microbiota/gut/brain axis [21]. Recent advances in the field have also confirmed that pesticides elicit detrimental effects on reproductive health and the endocrine profile, contributing to male and female infertility [23,24,25]. Importantly, the mutagenic and carcinogenic action of pesticides is well-documented [1,26,27]. Thus, pesticides pose significant risks to human health.

Identification of the cytotoxic molecular mechanisms associated with pesticides is of vital importance to expand our current understanding of their toxicological profiles and to develop approaches to reduce the negative impact of pesticides on human health. It should be emphasized that it is challenging to summarize the adverse effects of pesticides due to the significant chemical heterogeneity of this group of compounds. Fundamentally, pesticides are categorized into naturally occurring compounds and synthetic chemicals. In their turn, natural pesticides can be classified into plant-derived (e.g., pyrethrum, azadirachtin, rotenone, eugenol, thujone, or terthiophene) and mineral oils. The group of synthetic pesticides is more abundant and diverse and includes inorganic compounds (e.g., copper sulfate, iron sulfate, sulfur, or boric acid) and organic pesticides. The latter are categorized into organophosphates (malathion, fenitrothion, tempos, fenthos, dichrorvos, or pirimiphos), organochlorines (dichlorodiphenyltrichloroethane AKA DDT, benzene hexachloride AKA BHC, or gamma-hexachlorocyclohexane AKA lindane), carbamates (propoxur, bendiocarb, or carbaryl), neonicotinoids (acetamiprid, imidacloprid, or thiacloprid), pyrethroids (deltamethrin, cyfluthrin, bifenthrin, lembdacyhalothrin, or permethrin), diamides (chlorantraniliprole, cyantraniliprole, or flubendiamide), bipyridylium compounds (paraquat or diquat), avermectin compounds (abamectin or ivermectin) and other groups [3,9,28,29,30,31,32,33,34]. This chemical diversity determines a wide range of cellular and molecular targets for the biological action of pesticides [35]. Insecticides primarily target the nervous system, acting as acetylcholinesterase inhibitors, nicotine receptor agonists, voltage-gated sodium channel inhibitors, gamma-aminobutyric acid inhibitors, etc. [36,37]. Rodenticides can act as anticoagulants or mitochondrial oxidative phosphorylation uncouplers [38,39]. Fungicides can inhibit energy metabolism, microtubule assembly, or synthesis of fungal sterols [40]. Likewise, herbicides target specific plant metabolic pathways, for instance, inhibiting photosynthetic processes, as well as amino acid or lipid synthesis [41]. Thus, selectivity of pesticides is determined by the specific cellular mechanisms or signaling pathways they target.

At the same time, accumulating evidence suggests that despite different mechanisms of action and molecular targets, pesticides could share common cytotoxic mechanisms. In particular, Sule et al. suggested that oxidative stress triggered by excessive production of reactive oxygen species (ROS) and reactive nitrogen species (RNS) could be a common driving force of pesticide-induced cytotoxicity [42]. Moreover, a growing body of evidence indicates that pesticide-mediated toxicity is linked to inflammation induction [43,44]. Lopes-Ferreira et al. have recently summarized the available data and reported that exposure to a wide array of pesticides promotes upregulation of pro-inflammatory cytokines, including tumor necrosis factor-α (TNF-α), interleukin-1β (IL-1β), IL-6, IL-8, and many others, as well as cell damage accompanied by the release of damage-associated molecular patterns (DAMPs) and subsequent activation of Toll-like receptors (TLRs) and recruitment of immune cells like neutrophils and macrophages [44]. Importantly, cell demise associated with the liberation of DAMPs (known as alarmins) stimulates an adaptive immune response, which has resulted in the emergence of the concept of immunogenic cell death (ICD) [45]. According to the consensus guidelines on ICD, cells exposed to stress conditions can undergo immunologically active regulated cell death (RCD) [46]. ICD is hence linked to the ability of a cell undergoing RCD to emit immunogenic signals, which frequently (but not always) correlates with the lytic morphology of dying cells (this facilitates the release of immunogenic DAMPs) [47]. DAMP signaling has been reported to mediate immunogenicity of a wide spectrum of distinct RCDs, including ferroptosis, necroptosis, pyroptosis, and PANoptosis [47,48]. However, ICD cannot be oversimplified to be linked exclusively to DAMP signaling-promoting lytic RCD modalities. There is compelling evidence that apoptosis, in which membrane integrity is preserved, shows effects of ICD [49]. However, taken generally, apoptosis mostly manifests as anti-inflammatory or immunologically silent cell death [50]. In general, it is widely recognized that RCDs (currently over 20 distinct RCDs are reported) can occur with a wide range of morphological signs varying from totally necrotic to mostly apoptotic. Therefore, the effects on the immune system can range considerably in the following row: anti-inflammatory/tolerogenic/pro-inflammatory/immunogenic [51].

In this review article, we unveil RCD-associated effects of pesticides on the liver as a driving force of liver damage in hepatobiliary diseases. Herein, we report the cytotoxic molecular mechanisms associated with pesticide-induced liver hepatotoxicity and highlight how non-apoptotic RCDs shape liver pathology. Furthermore, we provide insights into the ability of different pesticides (with a primary focus on insecticides) to trigger distinct RCDs and underscore the role of pesticide-induced non-apoptotic RCDs in driving liver damage in the spectrum of liver diseases. Importantly, our review stresses the contribution of pesticide-induced cell death modes to inflammation and immunity regulation in hepatic pathology.

## 2. Liver Diseases and Pesticides: Toxicity Mechanisms That Are Not Related to Non-Apoptotic Regulated Cell Death Pathways

### 2.1. Liver Diseases: A Brief Overview

Liver diseases represent a heterogeneous group of disorders associated with injuries of hepatic parenchyma of dietary, bacterial, viral, toxic, hormonal, or immune etiology [52]. According to recent estimates, liver disease incidence demonstrates a growing trend and remains one of the leading causes of death globally [53]. For instance, over the last few years, non-alcoholic fatty liver disease (NAFLD), which is also referred to as metabolic dysfunction-associated steatotic liver disease (MASLD), and alcohol-associated liver disease (ALD) have significantly contributed to a global increase in the burden of chronic liver disease (CLD). Over 1 million deaths worldwide are annually linked to CLD that culminates in liver cirrhosis [54]. Importantly, MASLD is the most common CLD, which affects up to 30% of adults in the world. Its high incidence is associated with the pandemic of obesity and type 2 diabetes mellitus (T2DM). Both obesity and T2DM, developing as a result of metabolic alterations associated primarily with a hypercaloric diet and a sedentary lifestyle, are considered independent risk factors for the emergence of MASLD [55,56]. Additionally, chronic hepatitis B (CHB) and chronic hepatitis C (CHC) remain a medical challenge, especially in low- and middle-income countries [57]. Likewise, liver-associated genetic conditions like Wilson disease or alpha-1 antitrypsin deficiency and autoimmune hepatitis also cause a progressive deterioration of hepatic function and are classified as a type of liver pathologies that constitute CLD [58,59,60]. Chronic inflammation in CLD and hepatocellular injury create favorable conditions for cancer development, and hepatocellular carcinoma is currently considered a terminal step of CLD progression [61]. To sum up, CLD is linked to a permanent damage/regeneration cycle of hepatocytes associated with inflammation, which eventually promotes fibrosis and cirrhosis [62]. Therefore, the hepatotoxic factors that promote hepatocellular damage and boost liver inflammation aggravate CLD, accelerating the development of liver cirrhosis and altering the complex crosstalk between liver damage and repair. Importantly, hepatic inflammation is one of the key driving forces of damage to hepatocytes in CLD [63]. Continuous inflammation is tightly interconnected with oxidative stress, another crucial pathogenetic event in hepatic disorders [64,65]. At the cellular level, this pro-inflammatory microenvironment makes hepatocytes prone to cell death (in particular, apoptosis), stimulated in response to mitochondrial dysfunction, endoplasmic reticulum (ER) stress, lysosomal damage, ROS overproduction, and direct action of pro-inflammatory cytokines, triggering extrinsic apoptosis by binding to death receptors, etc. [66].

### 2.2. Pesticides Elicit Hepatotoxicity via Multiple Mechanisms

Converging lines of evidence indicate that pesticides elicit hepatotoxicity and can contribute to hepatocellular damage in multiple ways. In this section, we summarize the current knowledge of the impact of pesticide exposure on liver cells, highlighting the underlying molecular mechanisms. Pesticide exposure has been correlated with the increased risk of NAFLD [67,68], hepatocellular carcinoma [69,70,71], hepatitis B virus (HBV) infection [70], hepatitis C virus (HCV) infection [70], and elevation of circulating liver function markers such as aspartate aminotransferase (AST), alanine aminotransferase (ALT), alkaline phosphatase (ALP), or lactate dehydrogenase (LDH) [72,73,74,75]. Given the central role of the liver in the regulation of multiple metabolic processes and the fact that the liver acts as a central detoxification hub, including for pesticides [76,77,78], it is clear that the pesticide-hepatocyte interactions are of vivid interest to the research community.

#### 2.2.1. Metabolic Effects of Pesticides

**Figure 1:**
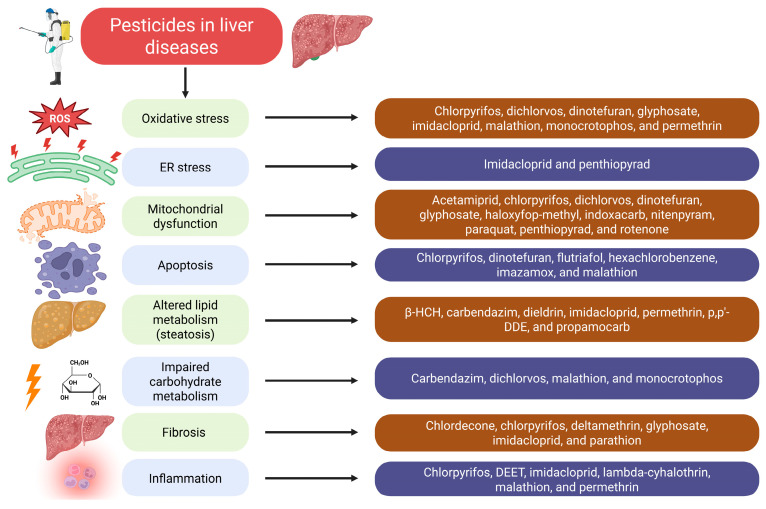
Pesticides drive liver diseases via induction of oxidative and ER stress, mitochondrial dysfunction, apoptosis, fibrosis, inflammation, or through altering lipid and carbohydrate metabolism in hepatocytes. Abbreviations: β-HCH, β-hexachlorocyclohexane; DEET, *N*,*N*-diethyl-*meta*-toluamide; ER, endoplasmic reticulum; p,p’-DDE, p,p’-dichlorodiphenyldichloroethylene; ROS, reactive oxygen species. Created with BioRender.com.

The liver plays a key role in homeostasis, suggesting that pesticides might modulate metabolism through their effects on hepatocytes. Indeed, metabolic alterations in the liver of animals exposed to different pesticides are well-documented [79,80,81]. Impaired lipid metabolism, which manifests by accumulation of triglycerides (TGs) in hepatocytes due to excessive lipogenesis and altered transport of TGs to the adipose tissue, impaired fatty acid β-oxidation, hypertriglyceridemia, etc., are hallmarks of NAFLD [82]. Notably, compelling evidence suggests that a wide array of pesticides representing different chemical groups promote lipid metabolism alterations typical for NAFLD (Figure 1). For instance, Bhatia and Venkitasubramanian demonstrated that dieldrin, an organochlorine pesticide, promoted TG accumulation in the liver, ascribed to enhanced lipogenesis [83]. Liu et al. reported that organochlorine pesticides such as p,p’-dichlorodiphenyldichloroethylene (p,p’-DDE) and β-hexachlorocyclohexane (β-HCH) were accumulated in the liver, stimulated mitochondrial dysfunction, and downregulated fatty acid β-oxidation, thereby altering energy metabolism of hepatocytes [84]. Aligned with the results presented above, mice administered imidacloprid, a systemic neonicotinoid insecticide, experienced reduced fatty acid oxidation in liver cells, as evidenced by downregulation of peroxisome proliferator-activated receptor alpha (PPARα) and peroxisome proliferator-activated receptor gamma coactivator 1-alpha (PGC-1α) [85]. Permethrin, a pyrethroid insecticide, stimulated fatty acid synthesis, lipogenesis, and TG accumulation in hepatocytes, simultaneously inhibiting fatty acid β-oxidation [86]. Khan et al. demonstrated that permethrin upregulated lipid metabolism enzymes via recruiting the KRAS/PPAR/GLUT signaling pathway in liver cells [87]. Carbendazim, a benzimidazole fungicide, increased circulating TG levels [88]. Conversely, propamocarb (a fungicide) downregulated the expression of genes involved in TG synthesis in the liver [89]. Nevertheless, taken together, it can be assumed that alterations of lipid metabolism triggered by a plethora of pesticides might link their exposure to NAFLD.

In addition to lipid metabolism, pesticides affect carbohydrate metabolism in liver cells, which also impairs the functions of hepatocytes (Figure 1). For instance, dichlorvos, an organophosphate pesticide, upregulated glucokinase expression, but glucokinase activity was found to be reduced [90]. Likewise, malathion, an organophosphate insecticide, upregulated glycogen phosphorylase and phosphoenolpyruvate carboxykinase in the rat liver, suggesting activation of gluconeogenesis and glycogen breakdown, thereby promoting hyperglycemia [91]. Of note, hyperglycemia is a well-documented risk factor for liver disorders [92]. Interestingly, the same effect was observed for monocrotophos, another organophosphate pesticide. It also promoted hyperglycemia (in diabetic rats) by stimulating glycogenolysis and *de novo* glucose synthesis in the liver [93]. Concomitantly, hyperglycemia in rats was induced by carbendazim [88]. Thus, carbohydrate metabolism disorders triggered by pesticides are also implicated in the overall hepatotoxicity of pesticides.

#### 2.2.2. Pesticides Promote Mitochondrial Dysfunction, Oxidative Stress, and Apoptosis in Liver Cells

Additionally, recent studies support the involvement of mitochondria as the target of hepatotoxic pesticides and underscore the importance of mitochondrial dysfunction in pesticide-induced hepatotoxicity (Figure 1). Chen et al. demonstrated that chlorpyrifos, endosulfan, fenpyroximate, paraquat, pendimethalin, rotenone, and tebufenpyrad promoted morphological mitochondrial changes and fragmentation [94]. Other studies have provided some insights into the molecular mechanisms of mitochondrial dysfunction induced by pesticides. For instance, neonicotinoid insecticides (dinotefuran, nitenpyram, and acetamiprid) promoted mitochondrial dysfunction of liver cells with subsequent ATP depletion via ROS generation and Ca^2+^ overload [95]. Likewise, dichlorvos stimulated mitochondrial dysfunction in liver cells due to Ca^2+^ influx and ROS accumulation. Importantly, mitochondrial dysfunction in dichlorvos-exposed liver cells was confirmed morphologically [96]. Haloxyfop-methyl and indoxacarb pesticides triggered mitochondrial damage in liver cells, which culminated in diminished oxidative phosphorylation [97]. Imidacloprid promoted damage to mtDNA [98]. Glyphosate-induced mitochondrial dysfunction was revealed to be linked to the inefficiency of the electron transport chain (ETC) and reduced ATP generation [99]. Similarly, chlorpyrifos, paraquat, and rotenone reduced the effectiveness of the ETC by blocking NADH dehydrogenase (complex I) [94]. Mitochondrial dysfunction was found to be linked with ER stress in hepatocytes of zebrafish co-exposed to cadmium and penthiopyrad, a fungicide that acts as a succinate dehydrogenase inhibitor [100]. Apart from ER stress induction, a wide array of pesticides altered proteostasis by inhibiting the 20S proteasome activity [94]. In line with this notion, ER stress markers like CHOP (C/EBP homologous protein), IRE1α (inositol-requiring enzyme 1 alpha), and XBP1 (X-box binding protein 1) were overexpressed in rats exposed to imidacloprid [101]. To conclude, mitochondrial damage and subsequent mitochondrial dysfunction are important mechanisms that determine the cytotoxicity of pesticides against liver cells.

As widely recognized, mitochondrial dysfunction leads to inappropriate oxidative phosphorylation and, therefore, ATP depletion, reinforces oxidative stress, and triggers intrinsic apoptosis [102]. Indeed, ROS generation with/without the associated depletion of the antioxidant system plays an important role in mediating the hepatotoxicity of monocrotophos [93], chlorpyrifos [103,104], dichlorvos [96], glyphosate [99], imidacloprid [105], permethrin [87], dinotefuran [95], malathion [106,107], and many others (Figure 1). ROS are multifaceted regulators of cellular processes, and oxidative stress mediated by their excessive generation is a well-known trigger of apoptosis [108]. This link has been clearly shown for pesticide-induced apoptosis in the liver. Dinotefuran triggered intrinsic apoptosis in liver cells linked to cytochrome c release in a ROS-dependent manner [95]. At the same time, apoptosis of liver cells induced by chlorpyrifos, an organophosphate insecticide, was linked to the recruitment of the JAK/STAT and MAPK pathways and ROS signaling [103,104]. Caspase-dependent mitochondrial pathways were also shown to be implicated in chlorpyrifos-induced apoptosis of QSG7701 human hepatocytes [109]. Flutriafol, a triazole fungicide, triggered apoptosis in HepG2 liver cells, which was associated with oxidative stress and mitochondrial dysfunction even against the background of the compensatory Nrf2 (nuclear factor E2 related factor 2) overexpression [110]. An herbicide, imazamox, promoted apoptosis of hepatocytes, confirmed morphologically and by caspase-3 cleavage in rats [111]. Malathion exposure in male Wistar albino rats promoted apoptosis of liver cells, as confirmed by the increased BAX/Bcl-2 ratio [106]. Furthermore, malathion upregulated *TP53*, *CASP3*, and *CASP9*, which ensured apoptosis of the liver cells of rats treated with this pesticide [107]. Hexachlorobenzene, an organochlorine pesticide, promoted both intrinsic cytochrome c-dependent apoptosis and Fas/FasL-mediated extrinsic cell death in rat liver [112]. Our analysis clearly unveils apoptosis as a driving force of pesticide-induced hepatotoxicity (Figure 1).

#### 2.2.3. Pesticides Trigger Hepatic Inflammation

As widely recognized, pesticides trigger hepatic inflammation (Figure 1). Morphological signs of inflammation were reported to develop in the rat liver following imidacloprid exposure [105]. Likewise, typical morphological features of hepatic inflammation, along with an increase in the content of pro-inflammatory TNF-α, IL-1β, and IL-6 in the hepatic tissue, were observed in mice administered chlorpyrifos [113]. Lambda-cyhalothrin, a pyrethroid insecticide, upregulated expression of TNF-α, IL-6, and IL-1β cytokines, an effect ascribed to excessive generation of ROS [114]. In rats, synthesis of TNF-α, IL-1β, IL-6, IL-12, and interferon (IFN)-γ in the liver was induced by imidacloprid. Notably, like in the case of lambda-cyhalothrin, ROS (generated by xanthine oxidase and myeloperoxidase) could contribute to cytokine production [115]. In Gulf War illness associated with exposure to several chemicals, including pesticides (*N*,*N*-diethyl-*meta*-toluamide, AKA DEET, and permethrin), the liver was reported to be infiltrated with D11b/c^+^ monocytes, and IL-6 levels were elevated [116]. Besides the mechanisms outlined above, it is worth mentioning that pesticides can directly activate pro-inflammatory signaling pathways in hepatocytes. As an example, chlorpyrifos promoted overexpression and activation of NOD-like receptor thermal protein domain-associated protein 3 (NLRP3), apoptosis-associated speck-like protein containing a caspase-recruitment domain (ASC), as well as the activation of the NF-кB signaling pathway in liver cells [103]. In human fetal L-02 hepatocytes, chlorpyrifos stimulated the NF-кB signaling pathway to increase cytokine production [104]. In an animal-based experiment, malathion upregulated TNF-α and NF-кB proteins in liver cells [106]. Additionally, overexpression of TLR2 and TLR4 in liver cells was demonstrated to be modulated by malathion [106] and chlorpyrifos [103]. Thus, a wide spectrum of pesticides triggers hepatic inflammation, which aggravates their hepatotoxic effects.

#### 2.2.4. Liver Fibrosis Is Linked to Pesticide Exposure

Importantly, exposure to pesticides has been linked to liver fibrosis, with glyphosate, chlorpyrifos, and parathion reported as major contributing pesticides [117] (Figure 1). Chlordecone, an organochlorine insecticide, induced fibrosis in a liver organoid-based model of MASLD [118]. In NAFLD patients, glyphosate promoted liver fibrosis [119]. Importantly, recent studies have shed light on the possible signaling pathways associated with liver fibrosis induction by pesticides. For instance, Han et al. reported that deltamethrin, a pyrethroid pesticide, triggered liver fibrosis, stimulating the TGF-β1 (transforming growth factor-β1)/Smad (small mother against decapentaplegic) signaling pathway [120], a crucial regulatory pathway whose activation results in upregulation of pro-fibrotic genes and synthesis of the components of the extracellular matrix [121]. This observation corroborates other findings emphasizing the crucial role of the TGF-β1/Smad pathway in imidacloprid-induced liver fibrosis [122]. Hexachlorobenzene also triggered TGF-β1 overexpression in liver cells [112]. At the same time, more studies should be conducted to widen our understanding of the links between pesticide exposure and liver fibrosis, with a focus on unlocking the signaling pathways mediating this relationship.

#### 2.2.5. Effects of Pesticides on Liver Cells Are Complex

Of note, OMICS studies support the complex, detrimental effects of pesticides on liver cells. For instance, a transcriptomic study performed by Jellali et al. showed that hepatic inflammation, steatosis, cell death, altered PPAR signaling, and impaired fatty acid metabolism were simultaneously detected following exposure to DDT and permethrin [123]. Likewise, a metabolomic animal-based study performed with the pesticide mixture (acetochlor, bromoxynil, carbofuran, chlormequat, ethephon, fenpropimorph, glyphosate, and imidacloprid) that reflected the common pattern of the pesticide environmental pollution in Brittany (France) showed simultaneous involvement of oxidative stress, mitochondrial dysfunction, impaired glucose and lipid metabolism in liver damage [124]. Therefore, the hepatotoxicity of pesticides might be multifaceted and suggests involvement of a wide array of cellular and molecular events.

A compelling body of evidence clearly demonstrates that a wide spectrum of pesticides might facilitate the development and progression of liver diseases through altering lipid and carbohydrate metabolism, triggering oxidative stress, ER stress, and mitochondrial dysfunction in liver cells, stimulating apoptosis, promoting fibrosis, and inflammation. At the same time, the impact of pesticide-induced non-apoptotic RCDs and their contribution to hepatic disorders remains underexplored and poorly summarized. In this review, we aim to fill this research gap and raise awareness of this mechanism to expand the landscape of the hepatotoxicological mechanisms associated with pesticides.

## 3. Cell Death Machinery and Non-Apoptotic Regulated Cell Death Pathways: Non-Apoptotic Regulated Cell Death Drives Liver Damage

Cell death is a fundamental phenomenon crucial for maintaining normal development and homeostasis in multicellular organisms [125]. Cells constantly sense a wide range of signals that regulate survival/demise. The balance between these competing signals either favors survival or provokes cell death. However, there is currently a strong consensus that the cell fate goes beyond this duality. Over recent decades, multiple forms of cell death have been identified, which are summarized in the most recent edition of the nomenclature suggestions and guidelines released by the Nomenclature Committee on Cell Death (NCCD) [51]. Fundamentally, the NCCD defines each cell death mode based on its unique biochemical characteristics, a genetically determined molecular machinery, and therefore a specific subset of regulatory and functional proteins. In contrast to the previously used morphological classification, this approach, based on specifying the signaling pathways governing cell demise, has significantly enriched our understanding of cell death biology. As a triumph of reductionism, this scientific route has offered multiple mechanistic insights and led to the identification of a great variety of distinct RCDs (e.g., intrinsic and extrinsic apoptosis, ferroptosis, autophagy-dependent cell death, parthanatos, necroptosis, entotic cell death, pyroptosis, lysosome-dependent cell death, NETotic cell death, etc.) [51]. However, from a broader and holistic perspective, these distinct RCDs are intimately interconnected. There is compelling evidence that molecular programs orchestrating distinct RCDs are tightly interrelated, forming a network of structured and synchronized signaling cascades whose interplay leads to a particular response from a cell [126,127,128,129]. This outcome manifests in death via one of the specific cell death modalities. At the moment, there is still an extensive gap in our understanding of the intracellular signaling molecules that can determine the switch between various types of RCDs. In particular, ROS are known to cause different RCDs mediating the crosstalk between various self-destructing cellular programs [126,130].

There is a growing interest in modulating RCDs to treat various diseases, including hepatic disorders. This interest is primarily linked to the fact that stress-induced cell death might alert the immune system, eliciting an inflammatory immune response. Herein, we highlight types of RCDs involved in the pathogenesis of liver diseases, delineating the importance of RCDs as drivers of pathological events in hepatic pathology.

### 3.1. RCDs Drive Liver Diseases

RCD pathways have been well-documented to navigate hepatic injury in liver diseases [131,132]. A wide array of studies have elucidated the implication of cell death pathways of hepatocytes and non-parenchymatous liver cells to the progression of hepatic disorders, including ALD (apoptosis [133], ferroptosis [134,135], necroptosis [136,137], pyroptosis [138], or autophagy-dependent cell death [139]), NAFLD (apoptosis [140], ferroptosis [141,142], necroptosis [143,144], autophagy-dependent cell death [145,146], pyroptosis [147,148], cuproptosis [149], or PANoptosis [150]), CHB (apoptosis [151], necroptosis [152], ferroptosis [153], pyroptosis [154], or autophagy-dependent cell death [155]), CHC (autophagy-dependent cell death [156], apoptosis [157,158], ferroptosis [159], pyroptosis [157,160], or necroptosis [161]), Wilson disease (cuproptosis [149,162,163], ferroptosis [162], or apoptosis [164]), hepatocellular carcinoma (PANoptosis [150], ferroptosis [165,166], necroptosis [165,166], or pyroptosis [165,166]), etc. To conclude, enhanced cell death of hepatocytes via different RCDs decreases the number of functional cells, compromises repair of damaged hepatic tissue, promotes replacement of functional hepatocytes with connective tissue (fibrosis), and fuels inflammation [131,132]. As amply outlined above, apoptosis, ferroptosis, necroptosis, and pyroptosis are the most documented and widely studied RCD pathways in liver pathology, contributing to a broad spectrum of liver diseases. Since the impact of both intrinsic and extrinsic apoptosis on liver diseases is abundantly described in a handful of good-quality reviews [167,168,169,170,171,172], the current article focuses on ferroptosis, necroptosis, and pyroptosis as the most commonly investigated and pathophysiologically relevant non-apoptotic RCDs that additionally share the features of ICD and therefore are important regulators of inflammation.

### 3.2. Ferroptosis Promotes Hepatic Damage

Ferroptosis is a cell death type mediated by ferrous iron, iron-dependent Fenton reaction-linked generation of ROS, and membrane phospholipid-derived peroxides [173]. Thus, the hallmarks of ferroptosis are alterations of iron, lipid, and redox metabolism [174]. Ferroptosis execution requires certain lipid- and redox-centric events, which include plasma membrane lipid peroxidation by ROS facilitated by glutathione peroxidase 4 (GPX4) and ferroptosis suppressor protein 1 (FSP1) depletion. Furthermore, the pro-ferroptotic imbalance between ROS and reduced glutathione (GSH), i.e., a higher ROS/GSH ratio, is also generated by the impaired system x_c_^−^, which transports cystine inside the cells to generate cysteine and then GSH [175]. Another important redox regulator whose status is critical for determining the sensitivity of cells to ferroptosis is Nrf2. This transcription factor is a master regulator of the cellular response to oxidative stress, which protects cells from oxidative damage by upregulating key antioxidant enzymes. Therefore, its deficiency depletes the antioxidant capacity of cells, favoring ferroptosis [176]. At the same time, ferroptosis is promoted by factors that contribute to intracellular iron release, ROS production, and lipid peroxidation. In particular, nuclear receptor coactivator 4 (NCOA4) upregulation promotes ferritinophagy, i.e., ferritin degradation, with subsequent iron overload that facilitates ferroptosis [177]. Likewise, iron accumulation that triggers ferroptosis is mediated by TFRC (transferrin receptors) upregulation, which favors Fe^2+^ import inside the cells [178]. Furthermore, upregulation of ACSL4 (acyl-CoA synthetase long-chain family member 4), LPCAT3 (lysophosphatidylcholine acyltransferase 3), and ALOXs (lipoxygenases) enables generation and peroxidation of fatty acids, thereby converting them into phospholipid hydroperoxides, crucial drivers of ferroptosis [174,179,180]. Although physiological and pathophysiological aspects of ferroptosis are not fully elucidated, there is accumulating evidence that ferroptosis is involved in tumor suppression, inflammation regulation, development, and aging [181].

Over recent years, ferroptosis has emerged as a multifaceted regulator of multiple pathological processes in the liver, which underscores its importance in the pathogenesis of hepatic pathology. There is a strong link between oxidative stress, impaired lipid metabolism, and ferroptosis induction in liver diseases [182]. Several studies have linked enhanced ferroptosis in the liver with steatohepatitis [183,184,185,186]. Peleman et al. summarized the ferroptosis-associated effects in the liver in MASLD, highlighting the cytotoxic role of 4-hydroxy-2-nonenal (4HNE), oxidized phospholipids, and DAMPs released from ferroptotic cells [187]. In particular, oxidized phospholipids were found to be implicated in alterations of energy metabolism in hepatocytes, with their subsequent apoptosis and accumulation of fats in them [187]. Likewise, ferroptosis-derived 4HNE promoted steatosis and mediated insulin resistance [188]. Indirectly, the role of ferroptosis in steatosis is supported by the ability of ferroptosis inhibitors like ferrostatin-1 to alleviate steatosis in animal models [183]. Besides steatosis, ferroptosis seems to promote liver fibrosis [189]. One suggested mechanism involved activation of hepatic stellate cells, which generate fibrosis-stimulating factors that cause fibroblasts to produce collagen and other ECM components. Moreover, hepatic stellate cells are converted to myofibroblasts to generate collagen I and other ECM structural proteins [190]. Activation of hepatic stellate cells is mediated by ROS associated with ferroptotic hepatocytes, ferroptosis-linked DAMPs that trigger cytokine production, and apoptosis of hepatocytes promoted by the generated pro-inflammatory microenvironment [191]. Furthermore, ferroptosis in the liver induces TGF-β generation by Kupffer cells, which acts as a master regulator of the formation of liver fibrous scar tissue [192]. Thus, accelerated ferroptosis in liver pathology links secondary apoptosis of hepatocytes, steatosis, fibrosis, and inflammation.

### 3.3. Necroptosis Is Implicated in the Pathogenesis of Liver Diseases

Necroptosis is frequently referred to as a regulated necrosis, since its morphological features are similar to those observed in necrosis, but it is orchestrated and executed by a clearly defined machinery comprising primarily MLKL (mixed lineage kinase domain-like pseudokinase), RIPK1 (receptor-interacting serine/threonine-protein kinase 1), and RIPK3 (receptor-interacting serine/threonine-protein kinase 3) [193]. Since necroptosis is characterized by necrotic morphology, its occurrence is accompanied by cell membrane perforation (pores are formed by MLKL polymers). Thereafter, the intracellular content is leaked from the necroptotic cells [194]. Necroptosis is initiated following activation of the TNF-α/TNFR1, FAS/FasL, TRAIL (TNF-related apoptosis-inducing ligand)/TRAIL-R (TRAIL receptor) signaling pathways, as well as in response to TLR3 or TLR4 signaling [195]. Importantly, necroptosis is tightly connected with apoptosis. Both pathways share the same triggers (such as death receptor signaling). Nowadays, it has been clearly documented that the apoptosis/necroptosis switch is dependent on the caspase-8 status. For necroptosis to occur, this caspase has to remain uncleaved (i.e., non-activated), while execution of apoptosis requires caspase-8 activation [196]. Post-translational modifications of the key components of the necroptotic pathway are widely recognized as critical for its signal transduction. RIPK1, RIPK3, MLKL, Fas-associated via death domain (FADD), caspase-8, and FLICE-like inhibitory protein (c-FLIP) are extensively regulated by ubiquitination and phosphorylation [197]. Necroptosis functions as a backup mechanism if cells fail to die by apoptosis. Furthermore, there is compelling evidence that its ability to trigger inflammation mediates its implication in the host defense against bacteria and viruses. Moreover, although necroptosis is reported to play a dual role in cancer, its tumor-suppressing and anti-tumor immunity-promoting functions are well-documented [198,199].

To date, necroptosis has been clearly demonstrated to modulate inflammation in liver diseases, especially in ALD and NAFLD. Regrettably, precise mechanisms remain elusive. Nevertheless, a handful of studies clearly link inflammation and fibrosis developing in response to inflammation-induced hepatocyte damage to DAMPs released by necroptotic cells [200]. Mohammed et al. underscored that enhanced necroptosis in liver diseases was attributable to excessive TNF-α and oxidative stress. As stated above, necroptosis was emphasized to maintain chronic low-grade inflammation and to induce fibrosis in the liver due to DAMP-mediated effects [201]. For instance, DAMPs released from necroptotic cells activate Kupffer cells, which reinforce inflammation and hepatocellular injury through the secretion of pro-inflammatory cytokines [202]. This hepatocellular damage triggers repair processes involving the generation of the ECM components [203]. Elucidation of the role of necroptosis in liver damage has shed light on the opportunity to inhibit it pharmaceutically in liver diseases to reverse fibrosis and mitigate inflammation [204]. Inflammation is tightly linked to steatosis, a hallmark of multiple liver diseases like ALD and NAFLD. For instance, RIPK1 and RIPK3 activation was shown to promote liver steatosis [136]. However, knowledge remains scarce regarding the possible molecular mechanisms involved. Importantly, necroptotic DAMPs have been postulated to promote progression of steatosis to fibrosis and then to hepatocellular carcinoma [205].

### 3.4. Pyroptosis Boosts Inflammation in Liver Diseases

Like necroptosis, pyroptosis has emerged as an inflammation-promoting cell death pathway involved in host defense and pathogen clearance. It was first reported for macrophages and was linked to the membrane rupture, which facilitated the release of IL-1β and IL-18 [206]. As an immunity-associated pathway, pyroptosis is canonically stimulated by DAMPs and pathogen-associated molecular patterns (PAMPs), which activate inflammasomes (NLRP3 is the most studied) [207]. Recruitment of the NLRP3 inflammasome promotes caspase-1 activation, which in pyroptosis has several critical substrates: gasdermin D (GSDMD), pro-IL-1β, and pro-IL-18. The cleaved GSDMD gets embedded into the cell membrane pores through which DAMPs and caspase-1-activated cytokines are released [208]. Besides this canonical pathway, pyroptosis occurs via caspase-4/5/11-dependent GSDME-associated non-canonical, caspase-3/8-mediated, or granzyme-mediated pathways [209]. As clearly outlined above, pyroptosis is primarily associated with the generation of pro-inflammatory cytokines and DAMPs; therefore, it prevents the spreading of pathogens and promotes anti-pathogen immunity [210].

A growing body of evidence supports a significant impact of pyroptosis and pyroptosis-associated immunogenic effects on liver diseases. Given the role of pyroptosis in anti-viral immunity, pyroptosis has been shown to be an important protective mechanism in HBV and HCV infections [211]. However, deregulated pyroptosis might be detrimental, promoting liver injury. As an example, pyroptosis-associated IL-1β and IL-18 are well-known drivers of hepatic inflammation and fibrosis [211]. Importantly, there is an inconsistency in the reports concerning the impact of IL-1β and IL-18 on steatosis. For instance, IL-1β stimulates TG accumulation in hepatocytes by driving lipogenesis, simultaneously inhibiting fatty acid oxidation [212]. Sim et al. also reported an association between elevation of circulating IL-18 levels and steatosis, as well as blood liver damage markers such as aminotransferases (ALT and AST) [213]. However, IL-18 was demonstrated to decrease TG deposits in the liver [214]. At the same time, both cytokines failed to trigger steatosis in a murine model [215]. However, it is widely accepted that, despite the possible discrepancies related to the effects on lipid metabolism and steatosis, IL-1β and IL-18 promote hepatocellular damage and destruction of hepatocytes [212,214,215]. Ample evidence suggests that IL-1 stimulates hepatic fibrogenesis. At the moment, our knowledge of the underlying mechanisms is limited. However, Gieling et al. linked pro-fibrotic effects of IL-1 with fibronectin synthesis and matrix metalloproteinase-9 (MMP-9) recruitment [216]. Moreover, there is some evidence that IL-1β promotes liver fibrosis by upregulating TGF-β [217,218]. Likewise, IL-18 was shown to directly activate hepatic stellate cells [219]. Thus, although the intricate mechanisms exploited by pyroptosis to affect liver homeostasis in liver pathology and to drive liver damage are far from being completely unveiled, the pyroptosis-linked contribution is primarily associated with IL-1β- and IL-18-mediated hepatocellular toxicity, steatosis, and fibrosis. A pro-inflammatory environment created by IL-1β and IL-18 might be favorable for hepatocellular carcinoma development [220].

**Figure 2:**
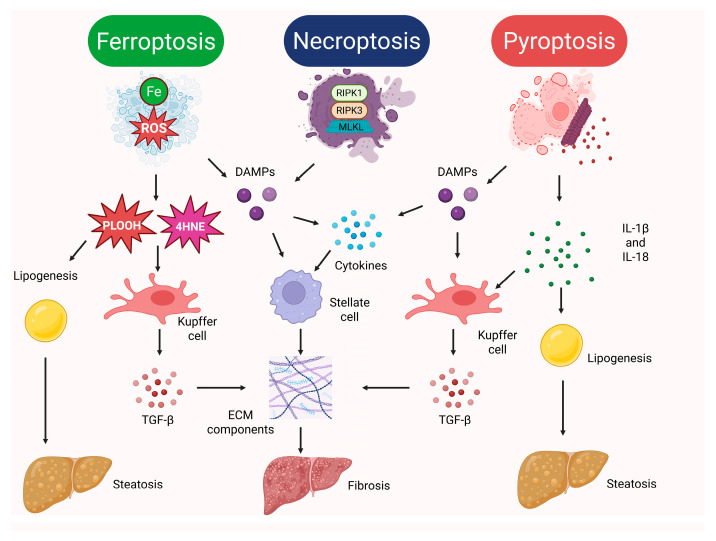
Key non-apoptotic regulated cell death pathways drive steatosis, fibrosis, and inflammation in liver diseases. DAMPs released from necroptotic, ferroptotic, and pyroptotic cells promote inflammation, cytokine production, and activation of Kupffer and stellate cells. Stellate cells produce collagen and other ECM components to trigger fibrosis. Ferroptotic cell-derived oxidized phospholipids and 4HNE stimulate lipogenesis to drive steatosis. Likewise, pyroptosis-related IL-1β and IL-18 induce steatosis. Abbreviations: 4HNE, 4-hydroxy-2-nonenal; DAMPs, damage-associated molecular patterns; ECM, extracellular matrix; IL-1β, interleukin 1β; IL-18, interleukin 18; TGF-β, transforming growth factor-β. Created with BioRender.com.

To conclude, although hepatocytes mostly die via apoptosis [131], other RCDs, such as ferroptosis, necroptosis, and pyroptosis, might be involved in shaping inflammation, contribute to further damage to hepatocytes, fuel steatosis and fibrosis in liver diseases (Figure 2), suggesting that their role in hepatic pathology should not be neglected.

## 4. Pesticides as Inducers of Non-Apoptotic Regulated Cell Death Modalities

Increasing evidence summarized in Table 1 suggests that induction of non-apoptotic cell death pathways like necroptosis, ferroptosis, and pyroptosis is a common mechanism of pesticide-induced toxicity. At the same time, guidance documents provided by the European Food Safety Authority (EFSA) and the 2025 Joint FAO/WHO Meeting on Pesticide Residues (JMPR) that are currently used for risk assessment of pesticides do not consider the ability of pesticides to trigger non-apoptotic RCDs [221].

### 4.1. Ferroptosis Induction Is an Important Mechanism of Pesticide Toxicity

Our analysis reveals that ferroptosis-mediated detrimental health effects of pesticides are currently the most studied, and ferroptosis contributes to pesticide-mediated nephrotoxicity [222,225], pulmonary toxicity [226,227,228,229,230], neurological damage [231,232,233,234,235,239,241,243,258], cardiotoxicity [236], reproductive dysfunction [224,237,238,242], intestinal injury [223], and immunotoxicity [240].

Of note, pesticides of different chemical classes are capable of inducing ferroptosis: neonicotinoids [222,223,224], bipyridinium compounds (paraquat) [226,227,228,229,230,231,258], rotenoids (rotenone) [232,233,234,235,236], organophosphates [238], and pyrethroids [239,240,241,242]. In line with a well-documented ability of pesticides to induce oxidative stress [42], redox imbalance was shown to be the key determinant of ferroptosis induction by imidacloprid [222,223], acetamiprid [224], avermectin [225], paraquat [226,227,228,230,258], co-exposure to paraquat and maneb [231], rotenone [232,233,234,235,236], glufosinate ammonium [237], chlorpyrifos [238], fenpropathrin [240], deltamethrin [241], and permethrin [242]. Although the sources of ROS in pesticide-induced ferroptosis are poorly studied, some evidence points to NADPH oxidase and mitochondria. In particular, ferroptosis-triggering ROS generation in cells exposed to a combination of paraquat and maneb was mediated by NADPH oxidase [231]. Likewise, redox imbalance might be aggravated by mitochondrial damage due to excessive generation of mitROS by injured mitochondria. For example, mitochondrial dysfunction associated with induced ferroptosis was reported for acetamiprid [224], paraquat [226,228,230,258], rotenone [234,235,236], glufosinate ammonium [237], or fenpropathrin [240]. Importantly, mitROS generation was directly demonstrated to contribute to rotenone-induced ferroptosis only [234]. Disrupted redox homeostasis in pesticide-induced ferroptosis can also be linked to a higher susceptibility of cells to lipid peroxidation associated with certain features of phospholipid metabolism. For instance, acetamiprid promoted ACSL4 upregulation, which resulted in an increase in the synthesis of ferroptosis-driving phospholipid peroxides [224]. Likewise, ACSL4- and ALOX12-mediated lipid peroxidation drove rotenone-induced ferroptosis [236]. ACSL4 mediated lipid peroxidation in glufosinate ammonium-induced ferroptosis [237]. Interestingly, tetrachlorobenzoquinone could induce ferroptosis without affecting the expression of ACSL4 [243]. Taken together, oxidative stress mediated by the generation of ROS and lipid peroxides is crucial for pesticide-induced ferroptosis.

Importantly, redox imbalance mediated by pesticides is further triggered by depletion of the antioxidant system. Indeed, oxidative stress induced by pesticides is frequently mediated by downregulation of the Keap1/Nrf-2 signaling pathway, which is a well-known master regulator of redox metabolism that upregulates antioxidant enzymes to combat oxidative stress and inhibit ferroptosis [259]. Nrf2 inactivation facilitated ferroptosis triggered by imidacloprid [222], paraquat [227], or rotenone [233]. At the same time, Nrf2 activators protected cells from ferroptosis induced by paraquat [226] and tetrachlorobenzoquinone [243]. Interestingly, rotenone could induce oxidative stress even when the Nfr2/HO-1 axis was activated [234]. Furthermore, Nrf2 activation in tetrachlorobenzoquinone-induced ferroptosis was reported to promote Fe^2+^ overload via upregulation of TFR1 and FTL [243]. Additionally, the GSH-based antioxidant defense following exposure to imidacloprid [222,223], acetamipirid [224], avermectin [225], paraquat [230,258], rotenone [232,234,235,236], glufosinate ammonium [237], chlorpyrifos [238], fenpropathrin [240], deltamethrin [241], or permethrin [242] was undermined by GPX4 and SLC7A11 downregulation, which decreased the intracellular content of GSH, contributing to ferroptosis. Interestingly, GPX4 and SLC7A11 downregulation by deltamethrin was found to occur in a p53-dependent manner [241]. The intracellular GSH levels decreased in paraquat-induced [226,258], chlorpyrifos-driven [238], deltamethrin-associated [241], and rotenone-triggered [233] ferroptosis. Likewise, the glutathione-independent link of the antioxidant system could be depleted in response to pesticides. For instance, rotenone downregulated superoxide dismutase (SOD) [232,235]. However, ferroptosis could be induced even if the compensatory GSH upregulation was observed, e.g., in glufosinate ammonium-induced ferroptosis [237]. Thus, ferroptosis induced by pesticides occurs as a response to an increase in the prooxidant/antioxidant ratio.

Evidently, iron accumulation promotes pesticide-induced ferroptosis. Iron accumulation was shown to be facilitated by FTH1 downregulation in imidacloprid-induced [222,223] and rotenone-associated [235] ferroptosis. Acetamiprid induced Fe^2+^ accumulation via TFR1 upregulation to ensure iron uptake by cells [224]. In line with this observation, TFR2 upregulation promoted bifenthrin-induced iron accumulation and subsequent ferroptosis [239]. Fenpropathrin triggered iron accumulation via TFR1 and NCOA4 upregulation and FTH1 downregulation [240]. Avermectin-triggered iron overload was linked to FTL and NCOA4 upregulation [225]. In paraquat-induced ferroptosis, Fe^2+^-regulating proteins were dysregulated as well (TFR1 upregulation and FTH1/FTL downregulation) [227]. Furthermore, converging lines of evidence demonstrate that the NCOA4/FTH ferritinophagy axis is responsible for iron overload in cells exposed to paraquat [228,258] and rotenone [234]. NCOA4-dependent ferritinophagy was implicated in iron-mediated damage and ferroptosis associated with glufosinate ammonium consumption [237]. Conversely, tetrachlorobenzoquinone-induced ferroptosis was revealed to be NCOA4-independent. However, it relied on the Nrf2/FTH1 axis [243]. At the same time, iron accumulation in cells co-exposed to paraquat and maneb was attributed to FPN-1 downregulation [231]. The same mechanism was reported for rotenone [233]. Taken together, impaired regulation of iron metabolism-associated proteins contributes to Fe^2+^ overload in pesticide-induced ferroptosis, which leads to Fenton reaction-mediated ROS production.

Accumulating evidence suggests that there is an extensive crosstalk between autophagy and ferroptosis [260,261]. Intriguingly, autophagy can fuel ferroptosis execution, acting as a ferroptosis enhancer [262]. The crosstalk between ferroptosis and autophagy was demonstrated for paraquat-induced cell damage, which was mediated by the acidification of lysosomes [228]. Additionally, ER stress contributed to paraquat-induced ferroptosis [229,230]. Rotenone simultaneously triggered ferroptosis, autophagy, and apoptosis [234]. Consistently, glufosinate ammonium promoted ferroptosis and autophagy, linked with the activation of the AMPK/ULK1 axis, which facilitated Fe^2+^ release due to ferritinophagy [237]. Clockophagy, a recently described ARNTL degradation-mediated mechanism of selective autophagy, was involved in the interplay between autophagy and ferroptosis in cells exposed to chlorpyrifos [238]. Thus, our analysis reveals that ferroptosis enhancement by autophagy machinery also occurs in pesticide-mediated cytotoxicity.

Regrettably, little is known about the links between ferroptosis and pesticide-induced inflammation. In particular, avermectin triggered ferroptosis and promoted renal inflammation associated with pro-inflammatory cytokines TNF-α, IL-1β, and IL-6 [225]. Likewise, ferroptosis in lungs induced by paraquat was accompanied by elevation of circulating IL-1β, IL-6, MCP-1, and TNF-α levels in rats [227]. Neuroinflammation associated with NF-κB-dependent microglial and astroglial activation and triggered by paraquat + maneb was attributable to ferroptosis-linked DAMPs [231]. Bifenthrin-mediated damage to neurons was associated with simultaneous induction of mitochondrial autophagy and ferroptosis [239]. Along with ferroptosis activation in lymphocytes, fenpropathrin induced the generation of IFN-γ, TNF-α, and IL-6 [240]. It is important to note that these studies have not provided robust evidence on the cause-and-effect relationships between ferroptosis (e.g., ferroptosis-related DAMPs) and inflammation following exposure to pesticides. More studies are required to shed light on the links between inflammation and ferroptosis in pesticide-induced toxicity.

### 4.2. Pesticides Induce Necroptosis

Necroptosis has emerged as an important toxicity mechanism [263]. In comparison to ferroptosis, our understanding of the role of pesticide-induced necroptosis in pathology remains elusive. However, accumulating evidence demonstrates that necroptosis triggered by pesticides might be involved in neurotoxicity and neurodegeneration [244,246,249], renal injury [245], cardiac dysfunction [247,248], and immunotoxicity [250,251]. Herein, we illuminate the mechanisms through which pesticides induce necroptosis. RIPK1/RIPK3/MLKL-dependent necroptosis was clearly demonstrated to be triggered by a wide spectrum of pesticides (Table 1), including rotenone [263], chlorothalonil [245], paraquat [246,247], dichlorvos [248], imidacloprid [250], and lambda-cyhalothrin [251]. Although acetamiprid was stated to trigger necroptosis, the involvement of the RIPK1/RIPK3/MLKL axis was not verified [249]. Additionally, paraquat triggered neither necroptosis nor ferroptosis in SH-SY5Y neuroblastoma and Neuro-2a cells, as evidenced by the inability of necrostatin-1 and ferrostatin-1 to save cells from paraquat-induced death and lack of changes in expression of RIPK1 and GPX4, suggesting that paraquat caused necrosis in these cell lines [264].

Our review underscores the importance of oxidative stress in the induction of necroptosis by pesticides, which is similar to ferroptosis stimulation by these compounds. Rotenone-induced necrosome formation was linked to ROS overproduction [263]. A similar contribution of ROS was reported to chlorothalonil-induced necroptosis [245]. Oxidative stress associated with ROS overproduction, enhanced lipid peroxidation, and antioxidant depletion promoted necroptosis induced by paraquat [246,247]. Dichlorvos-induced [248] and acetamiprid-driven [249] necroptosis was also upregulated by ROS. Additionally, ROS contributed to necroptosis induced by imidacloprid [250] and lambda-cyhalothrin [251]. Moreover, lambda-cyhalothrin exposure resulted in SOD and catalase downregulation [251].

Besides oxidative stress, other pathways are documented to mediate necroptosis in cells following pesticide exposure. For instance, ER stress was implicated in RIPK1 activation triggered by dichlorvos [248]. Likewise, ER stress was reported to accompany necroptosis triggered by acetamiprid [249]. Importantly, chlorothalonil promoted both apoptosis and necroptosis by upregulating the miR-15a/Bcl2-A20 axis via downregulation of Bcl2 and A20 (TNFAIP3) [245]. Another mechanism involved in necroptosis activation was demonstrated for paraquat and implied activation of the PTEN/PI3K/AKT pathway [246]. Importantly, autophagy inhibition enhanced dichlorvos-induced necroptosis, and sirtuin 1 (SIRT1) was found to reverse this necroptosis by promoting autophagy [248]. Acetamiprid-induced necroptosis in SH-SY5Y neural cells was not linked to NF-κB and TNF-α dysregulation [249]. Imidacloprid upregulated IL-6, IFN-γ, and TNF-α in lymphocytes, simultaneously triggering necroptosis [250]. Cai et al. clearly demonstrated that necroptosis triggered by lambda-cyhalothrin stimulated the ROS-mediated NF-κB signaling pathway in lymphocytes to upregulate TNF-α, INF-γ, IL-1β, IL-4, and IL-6 [251].

To date, our knowledge of necroptosis signaling cascades in pesticide-induced necroptosis is far from being unveiled. The RIPK1/RIPK3/MLKL axis is firmly established to mediate this necroptosis. However, its upstream regulators should be elucidated in further studies. At the moment, ROS seems to be the major contributor to necroptosis induction by pesticides.

### 4.3. Pyroptosis Triggered by Pesticides Contributes to Inflammation

Recent studies on pesticide-induced pyroptosis have unveiled its impact on kidney damage [222,253,254,265], neurological diseases [255,256], intestinal and pancreatic disorders [101,223,257], and immunotoxicity [252]. As shown in Table 1, a wide array of pesticides were shown to trigger pyroptosis: imidacloprid [101,222,223], fenpropathrin [265], thiacloprid [252], paraquat [253], malathion [254], rotenone [255,256], and propisochlor [257]. Imidacloprid-evoked pyroptosis was confirmed by identification of NLRP3, pro-IL-1β, mature IL-1β, GSDMD, and GSDMD-N upregulation with subsequent caspase-1 activation [222,223]. At the same time, NLRP3 inflammasome, caspase-1, and GSDMD mediated pyroptosis induced by fenpropathrin [265]. NLRP3, GSDMEA, and IL-18 upregulation indicated pyroptosis stimulation by thiacloprid [252]. Paraquat upregulated GSDMD [253]. NLRP3/caspase-1-mediated pyroptosis was triggered by malathion [254]. Rotenone-induced pyroptosis was linked to GSDMD, NLRP3, and ASC upregulation, as well as caspase-1 cleavage [255,256]. Propisochlor upregulated TLR4, NLRP3, caspase-1, and GSDMD to ensure pyroptosis [257]. To conclude, pyroptosis is commonly induced by pesticides.

Expectedly, ROS signaling was an important contributing factor to pyroptosis. ROS were involved in pyroptosis stimulation by fenpropathrin [265], rotenone [256], and imidacloprid [101]. Oxidative stress triggered by malathion was linked to SOD, catalase, and GSH depletion [254]. Thiacloprid promoted pyroptosis via triggering mitochondrial dysfunction associated with excessive mitROS generation and impaired mitochondrial bioenergetics (ATP depletion, reduced NADPH/NADP^+^ levels) [252]. In agreement with this observation, paraquat induced pyroptosis, promoting the production of mitROS [253]. Mitochondrial dysfunction was also involved in rotenone-driven pyroptosis [255]. Therefore, oxidative stress and mitochondrial dysfunction are implicated in pesticide-induced activation of the NLRP3/caspase-1/GSDMD signaling pathway.

There is also some evidence that pyroptosis driven by pesticides is regulated by alternative signaling cascades. The p38 MAPK signaling pathway promoted paraquat-evoked pyroptosis [253]. The TLR4/NF-κB pathway was implicated in imidacloprid-induced pyroptosis [222]. Downregulation of Parkin, an E3 ubiquitin ligase, mediated rotenone-induced pyroptosis [255]. Importantly, ER stress was linked to pyroptosis induction by imidacloprid [101]. Furthermore, imidacloprid-induced pyroptosis resulted in IL-1β release [101,223]. Likewise, the NF-κB pathway and IL-1β secretion accompanied fenpropathrin-induced pyroptosis [265]. Accumulating evidence shows that rotenone triggered the release of IL-1β and IL-18 as a result of pyroptosis [255,256]. Moreover, IL-1β and IL-18 were upregulated following exposure to propisochlor [257]. Notably, along with activation of pyroptosis and the release of pyroptosis-associated cytokines IL-1β and IL-18, thiacloprid upregulated IFN-γ, TNF-α, and IL-6 [252]. Likewise, not only IL-1β but also TNF-α and IL-6 were upregulated in malathion-induced pyroptosis [254]. Importantly, paraquat was shown to upregulate IL-1β in *Ctenopharyngodon idellus* fish kidney cells. However, no links that this IL-1β generation was related to pyroptosis were provided [246].

The crosstalk between pyroptosis and other RCDs in mediating the toxicity of pesticides is underexplored. Interestingly, imidacloprid-induced cytotoxicity was characterized by the interplay between ferroptosis and pyroptosis, since ferrostatin-1, a common ferroptosis inhibitor, inhibited NLRP3 and pro-IL-1β expression [222]. To better understand the mechanisms of pesticide-induced cytotoxicity, this issue should be further investigated.

Taken together, the findings summarized in this review suggest that pyroptosis might be a neglected enhancer of inflammation that mediates pesticide toxicity. Pyroptosis-related pro-inflammatory cytokines IL-1β and IL-18 are implicated in triggering or fueling pesticide-induced inflammation, contributing to various disorders.

## 5. Pesticides as Inducers of Regulated Cell Death Modalities in the Liver: Where Do We Stand?

**Figure 3:**
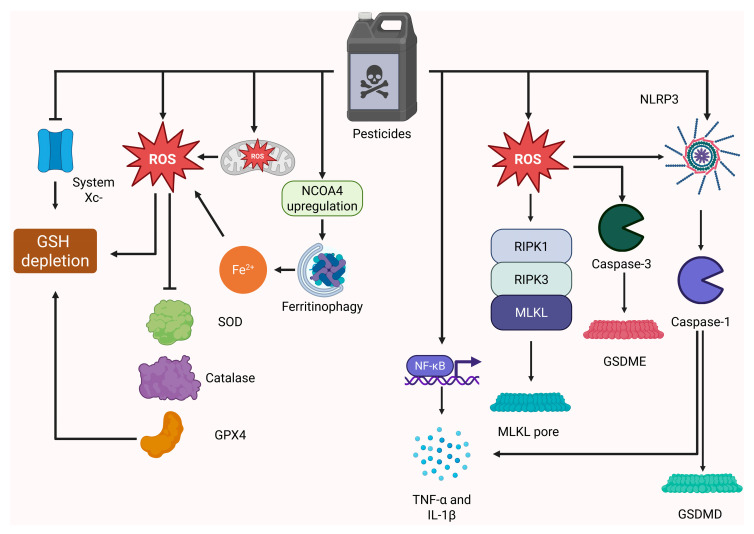
Pesticides promote ferroptosis, necroptosis, and pyroptosis in the liver, with ROS acting as key drivers of cell death. Ferroptosis induction in the liver is mediated by ROS production, mitochondrial dysfunction, Fe^2+^ accumulation (including due to NCOA4-mediated ferritinophagy), GPX4 downregulation, and system x_c_^−^ inhibition-associated GSH depletion. Additionally, ROS contributes to pesticide-induced necroptosis and pyroptosis via the RIPK1/RIPK3/MLKL, caspase-3/GSDME, and NLRP3/caspase-1 pathways. Abbreviations: GPX4, glutathione peroxidase 4; GSDMD, gasdermin D; GSDME, gasdermin E; GSH, reduced glutathione; IL-1β, interleukin 1β; MLKL, mixed lineage kinase domain-like pseudokinase; NCOA4, nuclear receptor coactivator 4-ferritin heavy chain; NF-κB, nuclear factor kappa-light-chain-enhancer of activated B cells; NLRP3, NOD-like receptor thermal protein domain-associated protein 3; RIPK1, receptor-interacting serine/threonine-protein kinase 1; RIPK3, receptor-interacting serine/threonine-protein kinase 3; ROS, reactive oxygen species; SOD, superoxide dismutase; TNF-α, tumor necrosis factor α. Created with BioRender.com.

Recent research has demonstrated the ability of pesticides to trigger non-apoptotic RCDs not only in liver parenchyma but also in non-parenchymatous cells. As summarized in Table 2, pesticides can trigger hepatic ferroptosis [229,266,267,268,269], necroptosis [270,271] and pyroptosis [272,273]. ROS might be of huge importance linking ferroptotic, necroptotic, and pyroptotic machinery in pesticide-induced liver damage (Figure 3).

### 5.1. Ferroptosis Triggers Pesticide-Induced Liver Damage

The most abundant experimental evidence supports the ferroptosis-triggering capacity of pesticides towards liver cells. Ge et al. demonstrated that paraquat deregulated the expression of ferroptosis-related genes in NCTC 1469 murine liver cells. In particular, *TFRC*, *SLC7A11*, and *CHAC1* were upregulated, while *ATF3* was downregulated. Thus, ferroptosis induction by paraquat in liver cells could be attributed to intracellular iron elevation, as well as system x_c_^−^- and ER stress-mediated GSH depletion [229]. Chlorantraniliprole, a bis-amide pesticide, induced ferroptosis in liver cells, as evidenced by downregulation of FTH1, GPX4, and upregulation of ACSL4 and NCOA4. Furthermore, chlorantraniliprole caused mitochondrial dysfunction linked with deregulation of mitochondrial fission/fusion, enhanced mitophagy, and accompanying mitROS generation in L8824 liver cells. Thus, mitROS contributed to chlorantraniliprole-induced oxidative damage to liver cells, further verified by enhanced lipid peroxidation, reduced activity of SOD, catalase, and glutathione peroxidase, as well as GSH depletion [268]. Expectedly, ROS and oxidative stress were crucial for the ability of other pesticides to trigger ferroptosis in the liver. Abamectin promoted lipid peroxidation, downregulated SOD and glutathione peroxidase, which triggered ferroptosis, as evidenced by GPX and SLC7A11 downregulation, as well as ACSL4 and p53 upregulation [266]. In line with these observations, ROS-mediated oxidative stress triggered by dichlorvos in BRL-3A liver-residing fibroblast-like cells was linked to Nrf2 and HO-1 downregulation and promoted SLC7A11 downregulation associated with ferroptosis [267]. Glyphosate induced ferroptosis in liver cells by promoting ROS generation, lipid peroxidation, GSH/GPX4 depletion linked with Nrf2 inactivation and ferritin deficiency-associated iron overload [269]. Thus, ferroptosis was demonstrated to be induced by abamectin, chlorantraniliprole, dichlorvos, glyphosate, and paraquat and linked primarily to the promotion of ROS generation.

Importantly, multiple studies have demonstrated that the ferroptotic machinery of liver cells is affected by pesticides with no specific verification of ferroptosis induction. For instance, although the induction of ferroptosis in the liver by lindane was not clearly shown, Kupffer cells of rats exposed to lindane experienced oxidative stress and iron accumulation, which might be indicative of ferroptosis induction [274]. Likewise, glyphosate promoted offspring liver injury in a rat-based model of perinatal exposure associated with hepatic iron accumulation, ROS overproduction, and NF-κB-dependent generation of TNF-α and IL-6 [275]. It should be noted that not only pesticides but also solvents used in pesticide manufacturing can trigger non-apoptotic cell death pathways in liver cells. For instance, ethyl carbamate triggered iron overload-associated ferroptosis in the liver of the treated mice by downregulating GSH and GPX4, as well as preventing Nrf2 activation [276,277].

Based on our analysis, the impact of pesticide-induced ferroptosis on inflammation in liver cells is underexplored. Direct consequences of ferroptosis induction by pesticides are poorly studied. However, simultaneously with ferroptosis stimulation, chlorantraniliprole upregulated pro-inflammatory TNF-α, IFN-γ, IL-1β, IL-2, IL-6, IL-17, IL-18, and downregulated anti-inflammatory IL-10 in hepatocytes [268]. More efforts should be undertaken to unlock this relationship, since this mechanism seems to be an important potential contributor to pesticide-induced liver injury.

### 5.2. Necroptosis Induction Is Linked to Pesticide-Mediated Hepatotoxicity

As outlined above, necroptosis is widely documented as a cell death modality in the liver. At the same time, our knowledge of pesticide-triggered necroptosis in hepatocytes and other liver cells remains elusive. In particular, Arora et al. demonstrated that deltamethrin-induced programmed necrosis in hepatocytes was dependent on activation of the RIPK1/RIPK3 axis in a caspase-independent manner (Figure 2). This pyrethroid insecticide induced necroptosis in a ROS-dependent fashion. Along with necroptosis induction, deltamethrin promoted NF-κB signaling and upregulation of TNF-α and inducible NO-synthase [270]. Similarly, glyphosate triggered RIPK1/RIPK3/MLKL-dependent necroptosis primarily due to alterations of the redox metabolism, which manifested by excessive production of ROS, lipid peroxidation, inactivation of catalase, SOD, and glutathione peroxidase, as well as a decrease in the content of GSH. Furthermore, PTEN-dependent inactivation of the PI3K/AKT signaling pathway was reported. Importantly, glyphosate upregulated TNF-α and IL-1β, along with necroptosis activation [271].

Thus, necroptosis expands the current landscape of the pathogenetic factors involved in pesticide-mediated hepatocellular toxicity. Although experimental data are scarce, necroptosis induction by pesticides might be linked to oxidative stress and is considered to correlate with pro-inflammatory properties of pesticides. Further studies could widen our knowledge of the contribution of necroptosis to liver damage mediated by pesticides and its role in aggravation of liver diseases by pesticides, especially stressing the necroptosis-associated immunogenic effects.

### 5.3. Pyroptosis Is a Driving Force of Pesticide-Induced Hepatic Injury

Pyroptosis, as a strongly pro-inflammatory type of RCD, has been hypothesized to be an important driver of inflammation in pesticide-triggered liver disorders. Indeed, currently available evidence supports that a few pesticides can induce pyroptosis. For instance, DDT, a widely used organochlorine pesticide, was capable of inducing caspase-3/GSDME pathway-mediated pyroptosis in normal liver cells, which was linked to oxidative stress and JNK activation. However, inflammation-fueling effects of DDT-induced pyroptosis (release of IL-1β and IL-18) were, regrettably, not investigated [272]. Furthermore, imidacloprid promoted NLRP3 and caspase-1 activation, IL-1β upregulation, and GSDMD cleavage in a P2 × 7-dependent manner in Kupffer cells [273]. The field experiences increasing interest in pyroptosis as a type of RCD that can drive inflammation and liver damage following pesticide exposure. Indeed, NLRP3 inflammasome-mediated pyroptosis might contribute to pesticide-induced hepatotoxicity. However, more mechanistic insights are required to elucidate the role of this RCD.

### 5.4. The Crosstalk Between RCD Modalities in Pesticide-Induced Liver Damage

It is worth mentioning that pesticides affect multiple cell death signaling pathways, which determines the diversity of RCD modalities triggered by them. Importantly, the crosstalk between ferroptosis and other RCDs was demonstrated in the liver following dichlorvos exposure. In particular, ROS triggered by dichlorvos promoted apoptosis along with ferroptosis. Of note, induction of ferroptosis and apoptosis required incomplete autophagy [267]. Likewise, pyroptosis and apoptosis are considered alternative RCDs in DDT-induced hepatotoxicity. GSDME knockdown switched DDT-induced pyroptosis in HL-7702 normal human liver cells to apoptosis, suggesting that apoptosis in this case acted as a backup mechanism. Importantly, ROS in these cells activated both caspase-3 activation and GSDME cleavage, which demonstrated their role in the regulation of both RCD modalities [272]. Likewise, glyphosate triggered both caspase-3-dependent apoptosis and RIPK1/RIPK3/MLKL-dependent necroptosis in the liver [271].

Taken together, there are extensive interactions between RCDs in the liver in response to pesticide exposure. They might be implicated in shaping the pesticide-mediated damage/repair of the hepatic parenchyma, remodeling the hepatic microenvironment, and orchestrating inflammation in pesticide-mediated liver disorders.

## 6. Concluding Remarks

In summary, our study confirms that pesticides carry significant health risks and sheds light on the underreported mechanisms that can drive their overall toxicity as a whole and hepatotoxicity in particular. The current experimental evidence clearly indicates that a wide spectrum of pesticides can trigger non-apoptotic RCDs in different tissues, which underscores the importance of this mechanism. In this review, we have focused on the role of RCDs, e.g., ferroptosis, necroptosis, and pyroptosis, in the emergence and progression of liver diseases associated with pesticide exposure. Accumulating evidence summarized in this review suggests that these emerging forms of RCD might be involved in promoting and orchestrating inflammation, liver tissue remodeling, steatosis, and fibrosis. Importantly, in this review, we have explored the specific signaling cascades that might be involved in the induction of particular non-apoptotic cell death pathways by pesticides or mediate the crosstalk between different RCDs. Our analysis has revealed the profound impact of pesticides on the cell death machinery of liver cells and the co-existence of several RCD modalities, highlighting the complex and context-dependent character of pesticide-induced hepatotoxicity. At the same time, as demonstrated in Table 3, our knowledge of pesticide-induced hepatotoxicity linked to non-apoptotic RCDs is limited, especially for naturally occurring pesticides. It is important to note that, in line with the generally recognized concept [42], ROS act as a critical modulator of pesticide-induced cell death pathways, acting not only as drivers of specific ferroptosis, necroptotic, and pyroptotic signaling cascades but also determining the crosstalk between these pathways (Figure 3). Thus, our review emphasizes the centrality of perturbed redox metabolism in pesticide-induced liver toxicity, underscoring its role in the regulation of cell fate and RCD-related effects. Like in the case of ROS, pesticide-induced mitochondrial damage and ER stress can trigger different types of RCD. However, it is difficult to identify which signaling pathways are critical for dictating the prevalent cell death modality.

As illustrated herein, not only can liver parenchymal cells undergo non-apoptotic RCDs in response to pesticides. There is some evidence that pesticides also stimulate non-parenchymal cells like Kupffer cells to die via ferroptosis, necroptosis, and pyroptosis. Little is known about the ability of pesticides to promote non-apoptotic RCDs in other non-parenchymal cells like lymphocytes, sinusoidal endothelial, or stellate cells. Although the links between pesticides, non-apoptotic RCDs, and inflammation in the liver are well-established, there is a lack of studies that directly investigate non-apoptotic RCD-mediated effects on inflammation, primarily those related to the release of DAMPs. This issue is of interest, since this mechanism can theoretically be a significant contributor to pesticide-mediated inflammation-associated hepatocellular injury. Furthermore, a growing body of evidence suggests that recently discovered and less widely characterized types of RCDs are also implicated in liver damage. For instance, parthanatos [131], cuproptosis [149], PANoptosis [150], entotic cell death [278], and others can regulate tissue damage in liver disorders. Regrettably, our knowledge of the ability of pesticides to alter signaling cascades involved in the execution of these RCDs is scarce. Elucidation of these aspects might further shed light on novel mechanisms of hepatocellular toxicity of pesticides.

Much has been done to improve our knowledge of pesticide-induced RCD-mediated hepatotoxicity and the research community has established that (i) pesticides of different chemical classes can trigger non-apoptotic RCDs (ferroptosis, necroptosis, and pyroptosis) of liver cells; (ii) pesticides can trigger non-apoptotic RCDs in both parenchymatous and non-parenchymatous liver cells; (iii) pesticide-induced non-apoptotic RCDs in the liver contribute to liver diseases regulating inflammation, liver tissue remodeling, steatosis, and fibrosis; (iv) ROS and oxidative stress are important driving forces of non-apoptotic RCDs induced by pesticides in the liver.

At the same time, the list of key remaining open questions in the field includes the following: (i) do pesticides trigger other non-apoptotic RCDs (e.g., PANoptosis, cuproptosis, parthanatos, disulfidptosis, methuosis, etc.) in the liver, and what is their contribution to hepatic damage? (ii) Do pesticides from the same chemical group share RCD-related patterns of hepatotoxicity? (iii) Are there any pesticides that can induce different RCDs simultaneously, and what are the factors that determine the selection of RCDs occurring in pesticide-exposed hepatocytes and non-parenchymatous liver cells? (iv) Which signaling cascades activated by pesticides govern the crosstalk between RCD pathways in the liver? (v) Are non-apoptotic RCD-associated effects in the liver (inflammation, liver tissue remodeling, steatosis, and fibrosis) pesticide-specific? It should be acknowledged that the current review has several limitations: (i) conclusions are made on a limited number of studies available, which weakens their strength; (ii) in addition to experimental studies in which RCDs were reliably verified, correlative studies were also taken into consideration; (iii) the lack of experimental data on pesticide-induced necroptosis and pyroptosis in the liver diminishes the scientific soundness of our conclusions; (iv) multiple studies analyzed in this research were performed on invertebrates limiting the width of conclusions and were characterized by low sample size, which restricted our capabilities of fully fledged risk assessment; (v) this study mostly focuses on insecticides with a limited attention to effects induced by fungicides and herbicides.

To conclude, non-apoptotic RCDs are significant pathogenetic events in pesticide-induced liver damage in a wide array of hepatic diseases. Ongoing studies will continue to provide novel insights into the links between pesticide exposure and RCD induction in the liver. These studies might contribute to the emergence of preventive strategies to reduce liver injury associated with pesticides. In particular, our analysis of the mechanisms involved in pesticide-induced ferroptosis in the liver has revealed that it seems to be promising to inhibit ferroptosis by ferrostatin-1 or liproxstatin-1 and iron chelators like deferoxamine. Given the role of Nrf2 in pesticide-induced ferroptosis, Nrf2 activation is also a viable strategy to inhibit ferroptosis and prevent hepatic damage mediated by pesticides. Although ferroptosis targeting is more feasible, specific necroptosis (e.g., necrostatin-1) and pyroptosis inhibitors (NLRP3 inflammasome inhibitors) might be of interest for further research. At the same time, antioxidants can be applied to inhibit all RCDs, which is underscored by the role of ROS in non-apoptotic RCDs induced by pesticides in liver cells.

## Figures and Tables

**Table 1:** Non-apoptotic regulated cell death pathways induced by pesticides.

Pesticide	Cell Lines or Living Organisms	RCD Modality	Molecular Mechanism	Effects	Reference
Imidacloprid, a neonicotinoid insecticide	C57/BL6 mice (in vivo)	Ferroptosis	Nrf2-deficient and oxidative stress-mediated ferroptosis of kidney cells	Nephrotoxicity	[222]
Imidacloprid, a neonicotinoid insecticide	Gut tissue of *Cyprinus carpio* (in vivo)	Ferroptosis	ROS- and iron-dependent ferroptosis	Intestinal toxicity	[223]
Acetamiprid, a neonicotinoid insecticide	Porcine oocytes	Ferroptosis	ROS-, iron accumulation-, and mitochondrial dysfunction-mediated ferroptosis	Oocyte quality deterioration	[224]
Avermectin, an insecticide	Freshwater carp (in vivo)	Ferroptosis	Oxidative stress-associated ferroptosis	Induction of nephrotoxicity and renal inflammation	[225]
Paraquat, a bipyridinium herbicide	A549 lung adenocarcinoma cells	Ferroptosis	Oxidative stress-associated and Nrf2 signaling pathway-mediated ferroptosis	Pulmonary toxicity	[226]
Paraquat, a bipyridinium herbicide	Sprague Dawley rats (in vivo)	Ferroptosis	ROS-dependent and Keap1/Nrf2 signaling pathway-mediated ferroptosis	Pulmonary fibrosis	[227]
Paraquat, a bipyridinium herbicide	A549 lung and BEAS-2B bronchial cell lines	Ferroptosis	Oxidative stress-associated and NCOA4/FTH ferritinophagy axis-dependent ferroptosis	Lung injury	[228]
Paraquat, a bipyridinium herbicide	TC-1 murine lung epithelial cells and TCMK-1 renal tubular cells	Ferroptosis	ER stress-associated ferroptosis	Pulmonary toxicity and nephrotoxicity	[229]
Paraquat, a bipyridinium herbicide	A549 human alveolar and RLE-6TN rat alveolar epithelial cells, as well as primary murine alveolar epithelial type II cells	Ferroptosis	ER stress-dependent PERK/eIF2α activation-mediated ferroptosis	Pulmonary toxicity	[230]
Co-exposure to paraquat, a bipyridinium herbicide, and maneb, a fungicide	Human neuroblastoma SH-SY5Y cells	Ferroptosis	NADPH oxidase-derived ROS-mediated ferroptosis	Dopaminergic neurodegeneration	[231]
Rotenone, a broad-spectrum insecticide	Primary cortical neurons and ICR mice (in vivo)	Ferroptosis	Iron-dependent ferroptosis	Exacerbation of intracerebral hemorrhage	[232]
Rotenone, a broad-spectrum insecticide	Human neuroblastoma SH-SY5Y cells and C57BL/6 mice (in vivo)	Ferroptosis	Sirtuin 1/Nrf2/ferroportin 1/GPX4 inhibition-associated ferroptosis	Dopaminergic neurodegeneration	[233]
Rotenone, a broad-spectrum insecticide	Human neuroblastoma SH-SY5Y cells	Ferroptosis	ROS- and mitochondrial dysfunction-mediated ferroptosis linked to autophagy and apoptosis induction	Dopaminergic neurodegeneration	[234]
Rotenone, a broad-spectrum insecticide	Murine brain organoids	Ferroptosis	Iron-, ROS- and mitochondrial damage-mediated ferroptosis	Neurotoxicity	[235]
Rotenone, a broad-spectrum insecticide	H9C2 rat cardiomyocytes	Ferroptosis	ROS-mediated ferroptosis	Cardiotoxicity	[236]
Glufosinate ammonium, a non-selective herbicide	TM3 and TM4 murine testicular cell lines and of Kunming mice (in vivo)	Ferroptosis	AMPK/ULK1-mediated ferritinophagy-dependent ferroptosis	Testicular toxicity	[237]
Chlorpyrifos, an organophosphate pesticide	TM4 murine testicular cell lines (Sertoli cells) and Sprague–Dawley rats (in vivo)	Ferroptosis	Clockophagy-dependent ferroptosis	Testicular toxicity	[238]
Bifenthrin, a pyrethroid insecticide	Parkin^−/−^ mice and C57BL/6 mice (in vivo)	Ferroptosis	Iron-dependent ferroptosis linked to activation of mitochondrial autophagy	Parkinson’s-like symptoms	[239]
Fenpropathrin, a pyrethroid insecticide	Lymphocytes of *Cyprinus carpio* (in vivo)	Ferroptosis	ROS-dependent, mitochondrial dysfunction-associated, and iron-dependent ferroptosis	Lymphotoxicity	[240]
Deltamethrin, a pyrethroid insecticide	HT22 neuronal cells and Wistar rats (in vivo)	Ferroptosis	p53-mediated dependent ferroptosis	Impaired hippocampal development in offspring as a result of maternal exposure	[241]
Permethrin, a pyrethroid insecticide	Zebrafish testes (in vivo)	Ferroptosis	Oxidative stress- and iron-dependent ferroptosis	Testicular damage	[242]
Tetrachlorobenzoquinone, a metabolite of the fungicide hexachlorobenzene	PC12 cells pheochromocytoma cells	Ferroptosis	Oxidative stress- and Nfr2 activation-dependent iron accumulation-associated ferroptosis	Neurotoxicity	[243]
Rotenone, a broad-spectrum insecticide	N2A neuroblastoma cells	Necroptosis	ROS-dependent RIPK1/RIPK3/MLKL-mediated necroptosis	Neurotoxicity and neurodegeneration	[244]
Chlorothalonil, a broad-spectrum organochloride fungicide	*Ctenopharyngodon idellus* fish kidney cell line	Necroptosis	ROS-dependent RIPK1/RIPK3/MLKL-mediated necroptosis linked to miR-15a/Bcl2-A20 downregulation	Nephrotoxicity	[245]
Paraquat, a bipyridinium herbicide	*Ctenopharyngodon idellus* fish kidney cell line	Necroptosis	Oxidative stress- and PTEN/PI3K/AKT-dependent RIPK1/RIPK3/MLKL-mediated necroptosis	Nephrotoxicity	[246]
Paraquat, a bipyridinium herbicide	Cardiomyocytes of C57BL/6J mice (in vivo)	Necroptosis	ROS-dependent RIPK1/RIPK3/MLKL-mediated necroptosis	Cardiac contractile dysfunction	[247]
Dichlorvos, an organophosphate insecticide	Cardiomyoblast H9c2 cell line and primary adult murine cardiomyocytes	Necroptosis	ROS- and ER stress-dependent RIPK1-mediated necroptosis	Cardiotoxicity	[248]
Acetamiprid, a neonicotinoid insecticide	Human neuroblastoma SH-SY5Y cells	Necroptosis	ROS- and ER stress-dependent necroptosis	Neurotoxicity	[249]
Imidacloprid, a neonicotinoid insecticide	Chicken lymphocyte lines	Necroptosis	Oxidative stress-linked, JNK/ERK/p38 MAPK-mediated caspase-8-dependent RIPK1/RIPK3/MLKL-associated necroptosis	Lymphotoxicity, impaired lymphocyte function	[250]
Lambda-cyhalothrin, a pyrethroid insecticide	Lymphocytes of *Cyprinus carpio* L. (in vivo)	Necroptosis	ROS-dependent RIPK1/RIPK3/MLKL-mediated necroptosis	Lymphotoxicity	[251]
Imidacloprid, a neonicotinoid insecticide	C57/BL6 mice (in vivo)	Pyroptosis	Ferroptosis-associated HMGB1/RAGE/TLR4/NF-κB signaling-mediated pyroptosis	Nephrotoxicity	[222]
Imidacloprid, a neonicotinoid insecticide	Gut tissue of *Cyprinus carpio* (in vivo)	Pyroptosis	NLRP3- and GSDMD-dependent pyroptosis	Intestinal toxicity	[223]
Imidacloprid, a neonicotinoid insecticide	Male Sprague–Dawley rats (in vivo)	Pyroptosis	IRE1α/XBP1/CHOP/NLRP3 signaling pathway-mediated caspase-1 activation	Pancreatic dysfunction induction	[101]
Thiacloprid, a pyrethroid insecticide	Lymphocytes of *Cyprinus carpio* (in vivo)	Pyroptosis	NLRP3- and GSDMD-dependent pyroptosis	Lymphotoxicity	[252]
Paraquat, a non-selective herbicide	HK-2 human proximal tubular cells and C57BL/6 mice (in vivo)	Pyroptosis	mitROS-dependent, p38 MAPK pathway-associated GSDMD-mediated pyroptosis	Acute kidney injury	[253]
Malathion, an organophosphorus insecticide	Wistar rats (in vivo)	Pyroptosis	NLRP3-dependent pyroptosis	Nephrotoxicity	[254]
Rotenone, a broad-spectrum insecticide	Mouse dopaminergic SN4741 neurons and C57BL/6 mice (in vivo)	Pyroptosis	Parkin/NLRP3-dependent pyroptosis	Dopaminergic neurodegeneration	[255]
Rotenone, a broad-spectrum insecticide	Murine hippocampal HT22 cells	Pyroptosis	ROS-mediated NLRP3/caspase-1/GSDMD-dependent pyroptosis	Dopaminergic neurodegeneration	[256]
Propisochlor, a chloroacetamide herbicide	Gut tissue of C57BL/6 mice (in vivo)	Pyroptosis	NLRP3/caspase-1/GSDMD-mediated pyroptosis	Intestinal inflammation and impaired intestinal barrier function	[257]

Abbreviations: AKT, protein kinase B; AMPK, AMP-activated protein kinase; CHOP, C/EBP homologous protein; eIF2α, eukaryotic initiation factor-2α; ER, endoplasmic reticulum; ERK, extracellular signal-regulated kinase; FTH, ferritin heavy chain; GPX4, glutathione peroxidase 4; GSDMD, gasdermin D; HMGB1, high-mobility group box 1; JNK, Jun N-terminal kinase; IRE1α, inositol-requiring enzyme 1 alpha; Keap1, Kelch-like ECH-associated protein 1; mitROS, mitochondrial reactive oxygen species; MLKL, mixed lineage kinase domain-like pseudokinase; NCOA4, nuclear receptor coactivator 4-ferritin heavy chain; NF-κB, nuclear factor kappa-light-chain-enhancer of activated B cells; NLRP3, NOD-like receptor thermal protein domain-associated protein 3; Nrf2, nuclear factor erythroid 2-related factor 2; p38 MAPK, p38 mitogen-activated protein kinase; PERK, protein kinase R-like endoplasmic reticulum kinase; PI3K, phosphoinositide 3-kinase; PTEN, phosphatase and tensin homolog; RAGE, receptor for advanced glycation end-products; RCD, regulated cell death; RIPK1, receptor-interacting serine/threonine-protein kinase 1; RIPK3, receptor-interacting serine/threonine-protein kinase 3; TLR4, Toll-like receptor 4; ULK1, Unc-51-like kinase 1; XBP1, X-box binding protein 1.

**Table 2:** Pesticide-induced non-apoptotic regulated cell death modalities in liver disease.

Pesticide	Cell Lines or Living Organisms	RCD Modality	Molecular Mechanism	Effects	Reference
Abamectin, a pesticide (glutamate-gated chloride channel activator)	Chinese mitten crab, i.e., *Eriocheir sinensis* (in vivo)	Ferroptosis	ROS-mediated ferroptosis in hepatopancreas	Damage to hepatopancreas	[266]
Glyphosate, a broad-spectrum herbicide	L02 human liver cells and BALB/c mice (in vivo)	Ferroptosis	Nrf2/GSH/GPX4 inhibition-linked ferroptosis	Hepatotoxicity	[269]
Paraquat, a non-selective herbicide	NCTC 1469 murine neonatal liver cells	Ferroptosis	ER stress-associated ferroptosis	Hepatotoxicity	[229]
Dichlorvos, an organophosphate insecticide	BRL-3A fibroblast-like liver-derived cells and Wistar rats (in vivo)	Ferroptosis	ROS-dependent, Nrf2/HO-1-associated ferroptosis	Hepatotoxicity	[267]
Chlorantraniliprole, a bis-amide pesticide	Grass carp L8824 liver cells	Ferroptosis	Oxidative stress- and mitochondrial dysfunction-dependent ferroptosis	Hepatotoxicity and inflammatory response	[268]
Deltamethrin, a pyrethroid insecticide	Primary hepatocytes derived from Wistar rats and Wistar rats (in vivo)	Necroptosis	ROS-dependent RIPK1/RIPK3-dependent programmed necrosis	Hepatotoxicity and inflammatory response	[270]
Glyphosate, a broad-spectrum herbicide	Grass carp L8824 liver cells	Necroptosis	ROS-dependent RIPK1/RIPK3/MLKL-dependent necroptosis	Hepatotoxicity and inflammatory response	[271]
Imidacloprid, a neonicotinoid insecticide	Murine liver Kupffer cells and C57BL/6 mice (in vivo)	Pyroptosis	P2×7-mediated, NLRP3- and GSDMD-dependent pyroptosis	Liver injury	[273]
DDT, an organochlorine insecticide	HL-7702 normal human liver cells	Pyroptosis	ROS/JNK/GSDME-mediated pyroptosis	Hepatotoxicity	[272]

Abbreviations: DDT, dichlorodiphenyltrichloroethane; ER, endoplasmic reticulum; GSDME, gasdermin E; GPX4, glutathione peroxidase 4; GSH, reduced glutathione; HO-1, heme oxygenase 1; JNK, c-Jun N-terminal kinase; Nrf2, nuclear factor erythroid 2-related factor 2; RCD, regulated cell death; ROS, reactive oxygen species.

**Table 3:** Pesticides from different chemical groups as inducers of non-apoptotic cell death pathways in the liver.

Pesticide Group	Ferroptosis	Necroptosis	Pyroptosis
Naturally occurring pesticides:			
plant-derived	N/A	N/A	N/A
mineral oils	N/A	N/A	N/A
Organic synthetic pesticides:			
organophosphates	Glyphosate [269] Dichlorvos [267]	Glyphosate [271]	N/A
organochlorines	N/A	N/A	DDT [272]
carbamates	N/A	N/A	N/A
neonicotinoids	N/A	N/A	Imidacloprid [273]
pyrethroids	N/A	Deltamethrin [270]	N/A
diamides	Chlorantraniliprole [268]	N/A	N/A
bipyridylium compounds	Paraquat [229]	N/A	N/A
avermectin compounds	Abamectin [266]	N/A	N/A

Abbreviations: DDT, dichlorodiphenyltrichloroethane; N/A, not available.

## Data Availability

The data that support this study are available from the corresponding authors, Talgat Medetbekov and Anton Tkachenko, upon reasonable request.

## Abbreviations

The following abbreviations are used in this manuscript:

4HNE	4-hydroxy-2-nonenal
β-HCH	β-hexachlorocyclohexane
ACSL4	Acyl-CoA synthetase long-chain family member 4
AKT	Protein kinase B
ALD	Alcohol-associated liver disease
ALOXs	Lipoxygenases
ALP	Alkaline phosphatase
ALT	Alanine aminotransferase
AMPK	AMP-activated protein kinase
ASC	Apoptosis-associated speck-like protein containing a caspase-recruitment domain
AST	Aspartate aminotransferase
BHC	Benzene hexachloride
c-FLIP	FLICE-like inhibitory protein
CHB	Chronic hepatitis B
CHC	Chronic hepatitis C
CHOP	C/EBP homologous protein
CLD	Chronic liver disease
DAMP	Damage-associated molecular pattern
DDT	Dichlorodiphenyltrichloroethane
DEET	*N*,*N*-diethyl-*meta*-toluamide
ECM	Extracellular matrix
eIF2α	Eukaryotic initiation factor-2α
ER	Endoplasmic reticulum
ERK	Extracellular signal-regulated kinase;
ETC	Electron transport chain
FADD	Fas-associated death domain protein
FSP1	Ferroptosis suppressor protein 1
FTH	Ferritin heavy chain
GPX4	Glutathione peroxidase 4
GSDMD	Gasdermin D
GSDME	Gasdermin E
HBV	Hepatitis B virus
HCV	Hepatitis C virus
HMGB1	High mobility group box 1
HO-1	Heme oxygenase 1
ICD	Immunogenic cell death
IFN	Interferon
IL	Interleukin
IRE1α	Inositol-requiring enzyme 1 alpha
JNK	Jun N-terminal kinase
Keap1	Kelch-like ECH-associated protein 1
LDH	Lactate dehydrogenase
LPCAT3	Lysophosphatidylcholine acyltransferase 3
MASLD	Metabolic dysfunction-associated steatotic liver disease
mitROS	Mitochondrial reactive oxygen species
MLKL	Mixed lineage kinase domain-like pseudokinase
MMP-9	Matrix metalloproteinase-9
NAFLD	Non-alcoholic fatty liver disease
NCCD	Nomenclature Committee on Cell Death
NCOA4	Nuclear receptor coactivator 4-ferritin heavy chain
NF-κB	Nuclear factor kappa-light-chain-enhancer of activated B cells
NLRP3	NOD-like receptor thermal protein domain-associated protein 3
Nrf2	Nuclear factor erythroid 2-related factor 2
p38 MAPK	p38 mitogen-activated protein kinase
PERK	Protein kinase R-like endoplasmic reticulum kinase
PGC-1α	Peroxisome proliferator-activated receptor gamma coactivator 1-alpha
PI3K	Phosphoinositide 3-kinase
p,p’-DDE	P,p’-dichlorodiphenyldichloroethylene
PPARα	Peroxisome proliferator-activated receptor alpha
PTEN	Phosphatase and tensin homolog
RAGE	Receptor for advanced glycation end-products
RCD	Regulated cell death
RNS	Reactive nitrogen species
ROS	Reactive oxygen species
RIPK1	Receptor-interacting serine/threonine-protein kinase 1
RIPK3	Receptor-interacting serine/threonine-protein kinase 3
Smas	Small mother against decapentaplegic
SOD	Superoxide dismutase
T2DM	Type 2 diabetes mellitus
TFR	Transferrin receptor
TG	Triglyceride
TGF-β1	Transforming growth factor-β1
TLR	Toll-like receptor
TNF-α	Tumor necrosis factor-α
TRAIL	TNF-related apoptosis-inducing ligand)
ULK1	Unc-51-like kinase 1
XBP1	X-box binding protein 1
